# Pharmacological Mechanism of NRICM101 for COVID-19 Treatments by Combined Network Pharmacology and Pharmacodynamics

**DOI:** 10.3390/ijms232315385

**Published:** 2022-12-06

**Authors:** Sher Singh, Ying-Fei Yang

**Affiliations:** 1 Department of Life Science, School of Life Science, College of Science, National Taiwan Normal University, Taipei 11677, Taiwan; 2 Department of Bioenvironmental Systems Engineering, National Taiwan University, Taipei 10617, Taiwan

**Keywords:** NRICM101, traditional Chinese medicine, protein–protein interaction, pharmacogenomics, biomarker, pharmacodynamics

## Abstract

Symptom treatments for Coronavirus disease 2019 (COVID-19) infection and Long COVID are one of the most critical issues of the pandemic era. In light of the lack of standardized medications for treating COVID-19 symptoms, traditional Chinese medicine (TCM) has emerged as a potentially viable strategy based on numerous studies and clinical manifestations. Taiwan Chingguan Yihau (NRICM101), a TCM designed based on a medicinal formula with a long history of almost 500 years, has demonstrated its antiviral properties through clinical studies, yet the pharmacogenomic knowledge for this formula remains unclear. The molecular mechanism of NRICM101 was systematically analyzed by using exploratory bioinformatics and pharmacodynamics (PD) approaches. Results showed that there were 434 common interactions found between NRICM101 and COVID-19 related genes/proteins. For the network pharmacology of the NRICM101, the 434 common interacting genes/proteins had the highest associations with the interleukin (IL)-17 signaling pathway in the Kyoto Encyclopedia of Genes and Genomes (KEGG) analyses. Moreover, the tumor necrosis factor (TNF) was found to have the highest association with the 30 most frequently curated NRICM101 chemicals. Disease analyses also revealed that the most relevant diseases with COVID-19 infections were pathology, followed by cancer, digestive system disease, and cardiovascular disease. The 30 most frequently curated human genes and 2 microRNAs identified in this study could also be used as molecular biomarkers or therapeutic options for COVID-19 treatments. In addition, dose–response profiles of NRICM101 doses and IL-6 or TNF-α expressions in cell cultures of murine alveolar macrophages were constructed to provide pharmacodynamic (PD) information of NRICM101. The prevalent use of NRICM101 for standardized treatments to attenuate common residual syndromes or chronic sequelae of COVID-19 were also revealed for post-pandemic future.

## 1. Introduction

With the catastrophes resulting from the emergence of the severe acute respiratory syndrome coronavirus 2 (SARS-CoV-2), the long coronavirus disease (COVID) experienced by patients with post-COVID symptoms is increasingly becoming a critical issue [1]. Long COVID has been found to be associated with a broad range of symptoms, among which fatigue, shortness of breath, altered smell/taste, muscle pain, joint pain, headache, cough, chest pain, and diarrhea are the most prevalent ones [2]. The dire situation of the lack of knowledge in the disease mechanisms of Long COVID and corresponding treatment options have led to a wide range of self-prescribed medications for the alleviation of Long COVID symptoms [3,4]. Among all kinds of developed or re-purposed medications for urgent treatments of patients post infection of COVID-19, traditional Chinese medicine (TCM) plays a distinct role in differentiating itself from modern Western medicine by taking overall approaches to look into syndrome differentiation, disease–syndrome combination, and treatment based on different stages of the disease. These characteristics enable the TCM to provide individualized treatments in patients with various physiologies for the achievement of treating both the manifestation of the disease and its cause [5,6].

While there have been no clinically approved medicines for COVID-19 treatments, TCM has proven its safety and effectiveness in treating COVID patients based on numerous clinical manifestations and evidence [7]. Treatment practices for COVID-19 showed that early intervention with TCM is effective in improving cure rates, shortening the courses of disease, delaying disease progressions, and reducing mortality rates. Furthermore, the early intervention of TCM was found to prevent the disease from transforming into more severe manifestations in patients with mild to moderate symptoms [6,8].

Taiwan Chingguan Yihau (NRICM101) is a novel TCM for which disruptions of disease progression and antiviral activities in COVID patients have been found since April 2020 [9]. The components of NRICM101 were designed based on a medicinal formula with a long history from almost 500 years ago for treatments of wind-cold-dampness or flu [9]. The bedside-to-bench study indicated that NRICM101 could disrupt disease progression through antiviral properties and act as a multi-target agent for COVID-19 prevention [10]. The NRICM101 has an ongoing clinical trial (https://clinicaltrials.gov/ct2/show/NCT04664049 (accessed on 24 November 2022)) and been approved for national use with Emergency Use Authorization (EUA) to treat patients with COVID-19 infections or Long COVID symptoms in Taiwan. With selections of plant-based and safe-to-use ingredients, NRICM101 has beneficial effects clinically and ensures its safety [10]. The safety of NRICM101 has also been approved with license keys for mass production by pharmaceutical companies in Taiwan. Import permissions or drug certificates of NRICM101 have also been issued in many countries including England, United States, Canada, South Africa, Singapore, Australia, and Thailand [11].

The primary NRICM101 study is a bedside-to-bench investigation in which pharmacological assays demonstrated the effects of NRICM101 in inhibiting the spike protein/angiotensin-converting enzyme 2 (ACE2) interaction, 3CL protease activity, viral plaque formation, and the production of cytokines interleukin (IL)-6 and tumor necrosis factor (TNF)-α [10]. Cheng et al. [12] also conducted an in silico study based on in vitro analyses on normal human lung fibroblast cells, evidencing the protective effects of NRICM101 against TNF-α/IL-1β-induced cell injury through c-Jun N-terminal kinase (JNK) and p38 mitogen-activated protein kinase (MAPK) signaling. In addition, a next-generation sequencing (NGS) analysis revealed that the inflammatory pathway, cell movement/infiltration of macrophages, and T-helper 1 (Th1)/T-helper 2 (Th2) immuno-regulation pathways were included in the pathways [12].

However, the previous NRICM101 study was simply conducted in human lung fibroblast cells, and a thorough analysis of pharmacogenomic network and pathway/disease analysis based on curated interactions of chemical contents of NRICM101, COVID-19, and human genomes has not been performed. We thus conducted network pharmacology analyses of NRICM101 to provide a comprehensive analysis of most associated pathways or diseases based on the curated gene sets/proteins of NRICM101 and COVID-19. The scarce understanding of the interactions of drug and responses of NRICM101 in the human body may constitute an obstacle for successful therapies because of the variations in different individuals. Pharmacogenomic analyses could overcome this predicament since they could provide detailed and personalized information of gene or protein interactions with the target chemicals of NRICM101 and related diseases in patients.

Therefore, with the aid of the abundant databases and powerful bioinformatics analysis tools, molecular mechanisms of NRICM101 and core biomarkers were identified to promote understanding of the molecular mechanisms of NRICM101 and COVID-19-associated genes or proteins. The pharmacogenomic analysis of NRICM101 in this study could facilitate the understanding of gene–chemical interactions at the molecular level, thereby improving individualization treatments of drugs and ensuring their efficacy and safety [13]. Variations in gene-encoding drug-metabolizing enzymes, receptors, or transporters could also provide insights to personalized therapies of COVID patients in the long-term perspective [4]. The pharmacogenomic-based disease analyses could also enable better understanding of modes of action of various symptoms in (Long) COVID patients and the provision of better treatment options.

## 2. Results

### 2.1. The TCM Chemicals of the 10 Herbs from NRICM101

**Figure 1:**
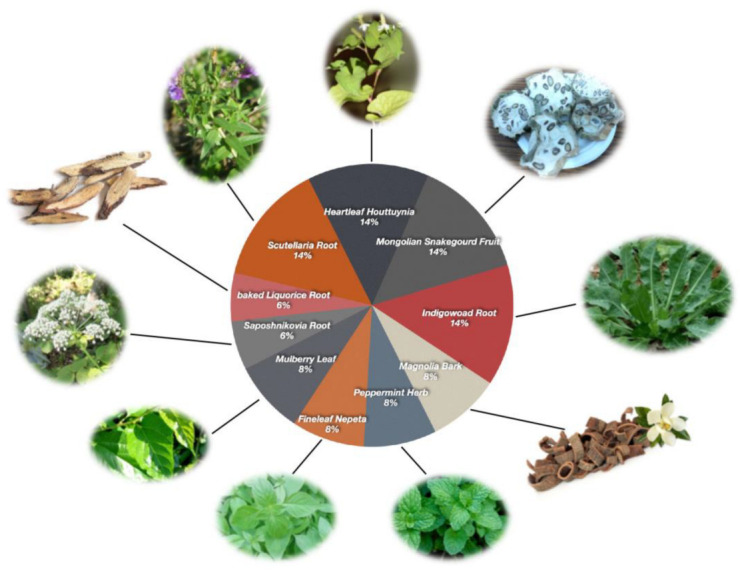
Compositions of the TCM formula: Taiwan Chingguan Yihau (NRICM101).

**Figure 2:**
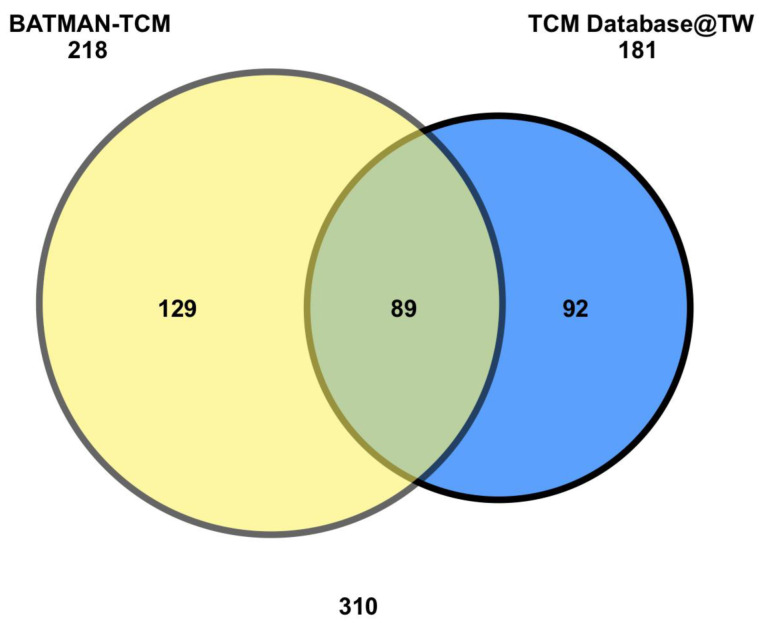
Venn diagram of NRICM101-10 herbs interacting chemicals. NRICM101 chemicals were found to interact with 218 and 181 unique chemicals, where 89 of them were in common.

The curated interactions between 10 herbs from NRICM101 and the Comparative Toxicogenomics Database (CTD) were obtained (Figure 1; Table 1). Subsequently, a total of 310 chemicals between the two TCM databases (Bioinformatics Analysis Tool for Molecular mechANism of Traditional Chinese Medicine (BATMAN-TCM) and TCM Database@Taiwan) were analyzed, resulting in 89 chemicals identified in both databases (Figure 2; Supplementary Table S1).

### 2.2. NRICM101 Chemicals and COVID-19 Related Gene/Proteins Analyses

Disease-associated genes were acquired from the Human Gene Database (GeneCards) and the Human Online Mendelian Inheritance Platform (OMIM) databases. The co-targeted genes/proteins as targets of both NRICM101 and COVID-19 were obtained from the CTD.

**Figure 3:**
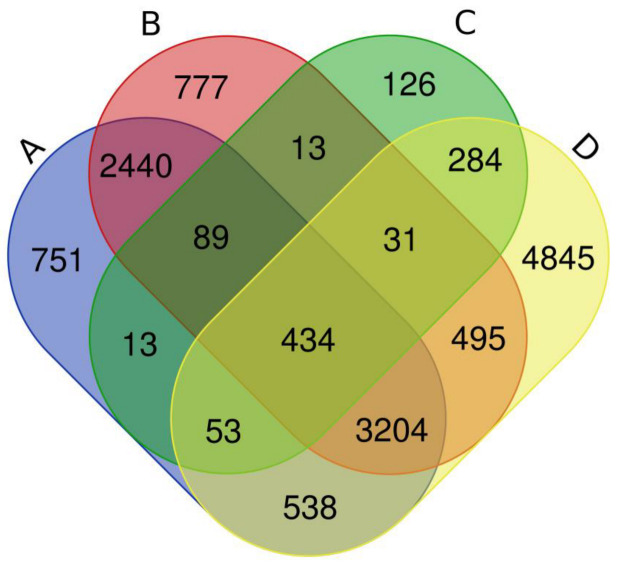
The four-way Venn diagram of interacted genes, and 434 of them were in common. A Venn diagram was constructed to identify overlapping genes in different gene sets. The four areas represent different related gene sets, where A represents BATMAN-TCM chemicals related 7522 gene/protein from CTD, B is TCM database@TW chemicals related 7483 gene/protein from CTD, C is COVID-19 related 1043 gene/protein from GeneCards, and D is COVID-19 related 9884 gene/protein from CTD. The cross areas indicate the overlapping common genes.

A total of 9884 co-targeted genes/proteins as targets of both NRICM101 and COVID-19 were found from the CTD as of 6 June 2022. There were 1043 COVID-19 related genes/proteins based on the analyses of the GeneCards human gene database. In addition, there were 7522 chemical-related human genes analyzed from the BATMAN-TCM and 7483 chemical-related human genes based on the TCM database@TW within the CTD (Figure 3; Supplementary Table S2). Finally, 434 common interactions were found between NRICM101 and COVID-19-related genes/proteins (Supplementary Table S3).

### 2.3. Gene Ontology, Pathway and Network Analyses

**Figure 4:**
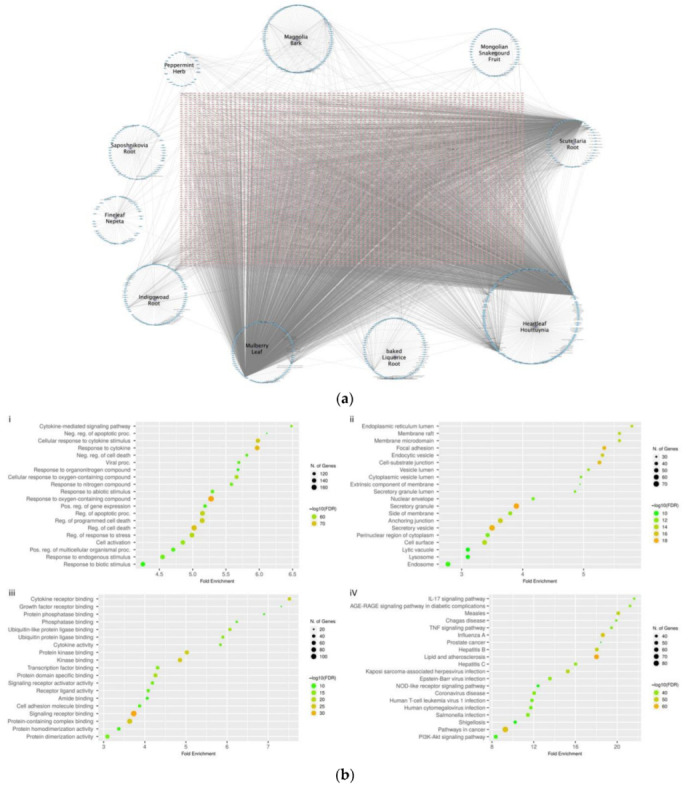

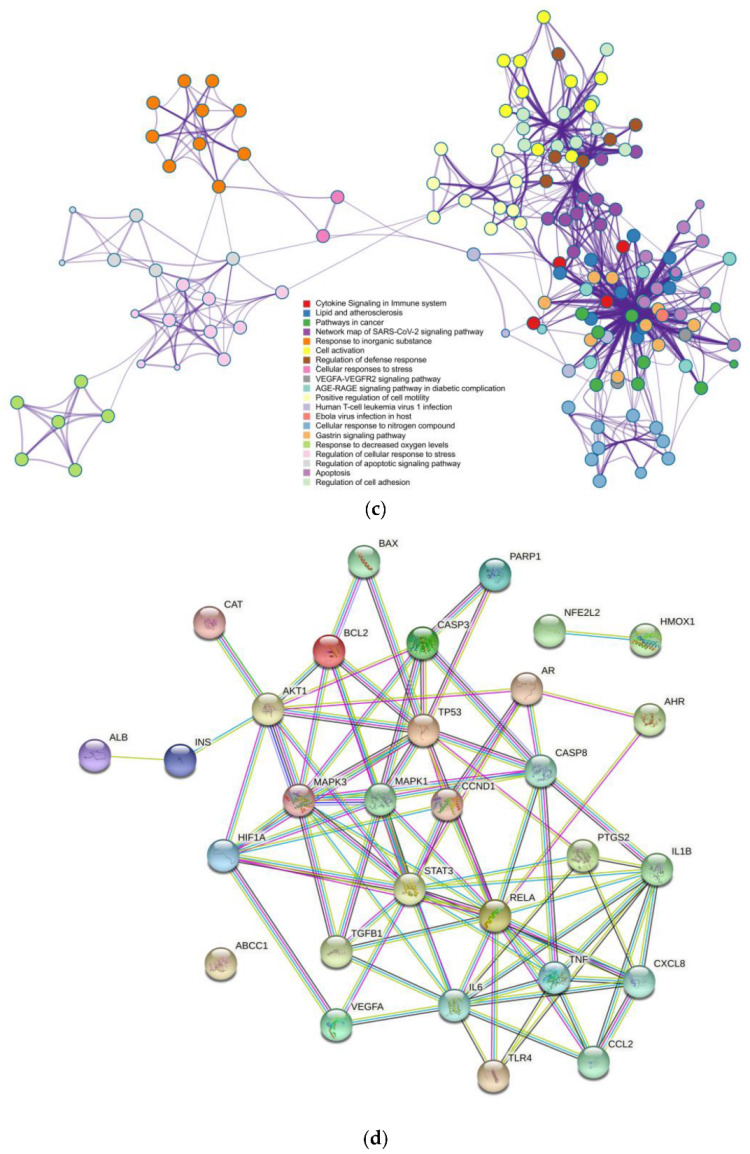
(**a**) Network pharmacology of herbs–chemicals–targets. (**b**) Gene ontology analyses of (i) biological process, (ii) cellular component, (iii) molecular function, and (iv) KEGG pathway enrichment analysis. (**c**) Ontology enrichment clustering network. (**d**) The PPI networks of the 30 most frequently curated genes/proteins. Network pharmacology of herbs–chemicals–targets relationships that contained the associations among herbs, targets, and chemical compounds of NRICM101. The pink nodes represent the module genes/proteins and the blue nodes represent the chemicals of herbs in NRICM101. GO term and KEGG pathway enrichment analysis of (i) biological process, (ii) cellular component, (iii) molecular function, and (iv) KEGG pathway for the target 434 genes. The gene ratios refer to the ratio of enriched genes to all target genes, and counts refer to the number of enriched genes. A complex clustering network was generated by the target 434 gene sets. It was visualized by Cytoscape with “force-directed” layout and with edge bundled for clarity. Terms with a similarity score > 0.3 were linked by an edge (the thickness of the edge represents the similarity score). One term from each cluster was selected to have its term description as shown in labels. The enrichment network visualization was shown with the intra-cluster and inter-cluster similarities of enriched terms. Cluster annotations were shown in color code. The PPI networks of the 30 most frequently curated genes/proteins of 434 gene set were further analyzed with the highest confidence interaction score of 0.9, evidence of network edges, 30 nodes, and 90 edges. The expected number of edges was 24, the average node degree was 6, and significant PPI enrichment *p*-value < 1.0 × 10^−16^.

The network pharmacology of herbs–chemicals–targets with NRICM101 was generated (Figure 4a). The 434 common interacting genes/proteins were completely analyzed for their gene ontology (GO), and the biological pathways (Figure 4b). In the (i) biological process (BP), the cytokine-mediated signaling pathway (GO:0019221) had the highest fold enrichment, followed by negative regulation of apoptotic process (GO:0043066), and cellular response to cytokine stimulus (GO:0071345). For (ii) cellular component (CC), endoplasmic reticulum lumen (GO:0005783) had the highest fold enrichment, followed by membrane raft (GO:0045121), and membrane microdomain (GO:0098857). For (iii) molecular function (MF), the order of fold enrichment was cytokine receptor binding (GO:0005126), growth factor receptor binding (GO:0070851), and protein phosphatase binding (GO:0019903). For the (iv) KEGG pathway, the order was IL-17 signaling pathway (hsa04657), AGE-RAGE signaling pathway in diabetic complications (hsa04933), and measles (hsa05162). It was also inferred by the CTD Perform analyses as set-based enrichment for collections in this investigation (Supplementary Tables S4 and S5).

The ontology enrichment clustering network was generated by 434 common gene sets (Figure 4c). Results showed that there were various pathways linked with the network map of the SARS-CoV-2 signaling pathway (dots in dark purple), among which cell activation, regulation of cell adhesion, regulation of defense response, and positive regulation of cell motility were clustered together. Other clusters of cytokine signaling in the immune system, gastrin-signaling pathway, lipid and atherosclerosis, apoptosis, pathways in cancer, AGE-RAGE signaling pathway in diabetic complications, and cellular response to nitrogen compound were more related. Other clusters of response to inorganic substances, regulation of apoptotic signaling pathway, regulation of cellular response to stress, and response to decreased oxygen levels could also be found (Figure 4c).

The 30 most frequently curated NRICM101 chemicals and COVID-19 related genes were sorted from the 434 common gene set by CTD with Homo sapiens and interaction count (Table 2). Results showed that the COVID-19 related tumor necrosis factor (TNF) had the highest association with the 30 most frequently curated NRICM101 chemicals, followed by caspase 3 (CASP3), interleukin 1 beta (IL1B), insulin (INS), AKT serine/threonine kinase 1 (AKT1), BCL2 associated X, apoptosis regulator (BAX), BCL2 apoptosis regulator (BCL2), NFE2-like bZIP transcription factor 2 (NFE2L2), and interleukin 6 (IL6) (Table 2). Functional enrichment analyses for the collection of the 30 most frequently curated genes, gene ontology (GO) pathways, and networks were fully analyzed (Supplementary Tables S6–S8).

The GO molecular functions of 434 unique NRICM101 chemicals and COVID-19 interacting genes and the 30 most frequently sorted unique gene sets were analyzed, and their molecular functions of GO terms are presented in Supplementary Tables S4 and S6. There were, respectively, 37 and 39 functions out of the top 50 ones from the CTD and STRING involved with small molecular bindings. It was also found that 44 GOs in the molecular functions identified by the two methods were the same (Supplementary Tables S4 and S6). It should be noted that many of the GOs and pathways of the 434 unique NRICM101 chemical and COVID-19 interacting genes were found to be quite similar to those of the 30 most frequently selected unique gene sets (Supplementary Tables S4 and S6). The top 10 pathways were involved in the immune system, innate immune system, cytokine signaling in the immune system, and virus infection pathways, while 46 of the top 50 biological pathways were the same (Supplementary Tables S5, S7 and S8), indicating that these 30 unique subsets of the gene sets likely represent the overall interacting genes of the 434 genes responding to both COVID-19 and NRICM101. The PPI networks of the 30 most frequently curated genes/proteins of 434 gene sets were further analyzed with the highest confidence interaction score of 0.9, evidence of network edges, 30 nodes, and 90 edges. The expected number of edges was 24, the average node degree was 6, and significant PPI enrichment *p*-value was < 1.0 × 10^−16^ (Figure 4d).

### 2.4. Disease Analysis

**Figure 5:**
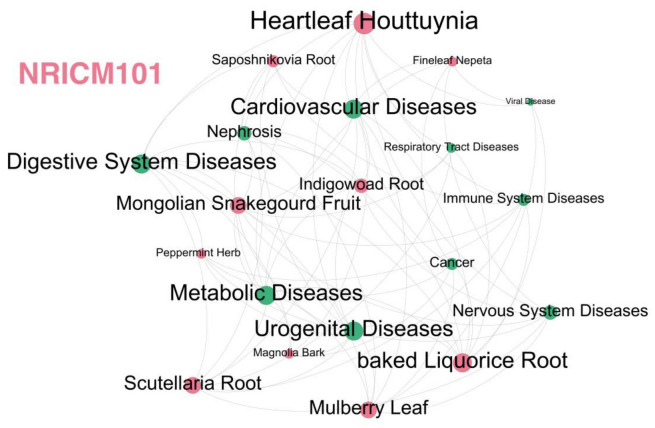
The herb–meridian disease network of NRICM101. Dots in red and green represent herbs and diseases, respectively. The NRICM101 includes 10 herbs of Scutellaria Root (heat-clearing and damp-drying medicine), Heartleaf Houttuynia (heat-clearing and detoxifying drugs), Mongolian Snakegourd Fruit (heat-clearing and phlegm-resolving medicine), Indigowoad Root (heat-clearing and detoxifying drugs), Magnolia Bark (dampness medicine), Peppermint Herb (Diffuse wind-heat medicine), Fineleaf Nepeta (post apothecary), Mulberry Leaf (heat-clearing medicine), Saposhnikovia Root (Healing wind, dispersing muscle surface wind evil medicine), and Baked Licorice Root (Qi tonic). The NRICM101 has the functions of detoxification and dampness, clearing heat and relieving asthma, and enhancing the body’s immunity.

The 30 and 434 NRICM101 chemicals and COVID-19 interacting gene sets were fully analyzed for their human diseases inferred by the CTD perform analyses as set-based enrichment for collections in this investigation (Table 3 and Supplementary Table S9). Many of the 434 unique NRICM101 chemical and COVID-19 interacting genes for enriched diseases analyses were found to be very similar to those in the most frequently selected 30 unique gene sets, and 24 of the TOP50 diseases in enriched diseases from 434 genes were identical in top 30 enriched diseases from the 30 most frequently curated gene set, indicating that subsets of these 30 unique gene sets likely represent the overall enriched diseases of 434 genes that respond to COVID-19 and NRICM101 (Table 3 and Supplementary Table S9), which contain digestive system disease, vascular diseases, cardiovascular disease, cancer, nervous system disease, digestive system disease, immune disease, respiratory tract disease, metabolic disease, and reproductive system disease that respond to COVID-19 and NRICM101 (Figure 5).

Through the enriched disease analysis, the most relevant diseases were pathology, cancer, digestive system disease, cardiovascular disease, nervous system disease, immune system disease, respiratory tract disease, metabolic disease, urogenital disease, skin disease, viral disease, and nephrosis, etc. These 10 herbs have binding forces to spike proteins of the SARS-CoV-2, and the remaining herbs of NRICM101 are mainly reflected in the effects on immunity, inflammation, and related signaling pathways (Figure 5). The functional enrichment analyses for disease–gene associations of the 30 most frequently curated NRICM101 chemicals and COVID-19 related genes were also analyzed for the STRING database (Supplementary Table S10).

### 2.5. Target Identification of Genes

The potential target miRNAs of the 30 most frequently curated genes were predicted using the miRabel database [14]; the 10 target genes (CXCL8, AHR, MAPK1, PARP1, AR, STAT3, VEGFA, TGFB1, ALB, and TLR4) of the hsa-miR-31-5p microRNA and the 14 target genes (AKT1, AHR, RELA, MAPK3, MAPK1, TP53, PARP1, HMOX1, STAT3, VEGFA, TGFB1, CCL2, CASP8, and ABCC1) of the hsa-miR-1275 were found from the 30 most frequently curated genes set individually (Table 4).

### 2.6. Pharmacodynamics of NRICM101

**Figure 6:**
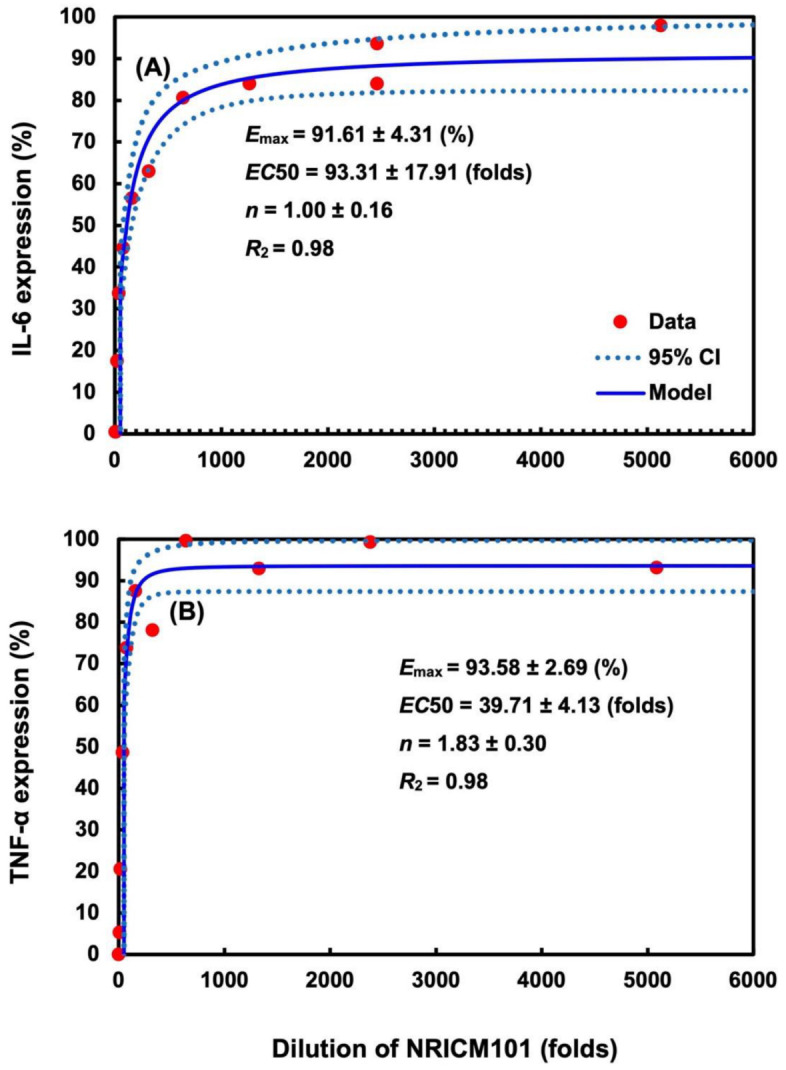
Pharmacodynamics analysis of relationships between cytokine expressions of (**A**) IL-6 and (**B**) TNF-α (%) and dilution folds of NRICM101. Dose–response profiles of NRICM101 with different dilution folds and corresponding cytokine inhibitions (IL-6 or TNF-α expression) in cell cultures of murine alveolar macrophages were established by adapting the experimental data [10]. A three-parameter Hill model was applied to fit the experimental data describing the relationship between the dilution folds of NRICM101 and increments of IL-6 or TNF-α expression (%) [10].

To more deeply explore the pharmacological effects of NRICM101, the relationships between cytokine expressions of IL-6 or TNF-α (%) and dilution folds of NRICM101 were constructed by applying the Hill-based dose–response profiles (Figure 6; Supplementary Table S11). The estimated dilution folds leading to half of the expressions (*ED*50) of IL-6 and TNF-α were 93.31 ± 17.91 (mean ± standard error (S.E.)) and 39.71 ± 4.13, approximately corresponding to the NRICM101 doses of 0.31 and 1.04 mg mL^−1^, respectively (Figure 6; Supplementary Table S11). The fitted Hill coefficients (*n*) were 1.00 ± 0.16 (*r*^2^ = 0.98, *p* < 0.001), 1.83 ± 0.30 (*r*^2^ = 0.98, *p* < 0.001 for IL-6 and TNF-α, respectively. Maximum effects of IL-6 and TNF-α expressions (*E*_max_) were found to be 91.61 ± 4.31 and 93.58 ± 2.69 (%) (Supplementary Table S11).

## 3. Discussion

The lack of effective treatments for COVID-19 symptoms and scarce knowledge of adverse drug reactions (ADRs) have been a critical issue since the pandemic. Except for clinical manifestations, evidence for effective treatments or recommendations for prescribing still remain controversial due to their complex interactions in human body [4]. Pharmacogenomics (pharmacogenetics) is one of the most potential tools to deeply explore human gene/protein interactions with specific molecules in the drug candidate and explain variations in patients’ outcomes. TCM has established its value and lasting contributions in combating COVID-19 during the pandemic era based on multiple pre-clinical or clinical evidence [15]. Therefore, due to the increasing use of TCM in many geographic regions, comprehensive understanding of the underlying drug mechanisms in treating COVID-19 patients through pharmacogenomic analyses is of great importance.

Although different TCMs are composed of different herbs, it was suggested that some formulas of TCM have similar core targets against COVID-19 [16]. Additionally, mechanisms for TCM treatments in patients with mild symptoms were revealed to be antiviral and symptom relieving. For more severe or critical cases, antiviral activities, protection of target organs, and cytokine storm inhibitions were suggested [16]. Tsai et al. [10] also confirmed in a bedside-to-bench study that NRICM101 could disrupt disease progressions in COVID patients through its antiviral and anti-inflammatory properties, indicating that NRICM101 could be promising for treatments of both acute COVID-19 and chronic sequelae. In accordance with the results in this study, inhibitions of spike protein/angiotensin converting enzyme 2 (ACE2) interaction, viral plaque formation, 3C-like protease (3CL^pro^) activity, and the production of cytokines interleukin (IL)-6 and tumor necrosis factor (TNF)-α were also demonstrated in pharmacological assays. Additionally, Cheng et al. [12] further supported our study through an RNA sequencing transcriptional profile analysis that there were evident changes in mRNA expressions, among which 213 genes were up-regulated and 118 genes were down-regulated in TNF-a/IL-1b injured lung cells treated with NRICM101. On the other hand, higher cell viability was observed in TNF-a/IL-1b injured lung cells, whereas attenuation effects (decreased levels) were found in c-Jun N-terminal kinase (JNK) and mitogen-activated protein kinase (MAPK) activities in higher dose treatments.

It was found that the top five pathways regulating interactions between the NRICM101 and SARS-CoV-2 were immune system, followed by innate immune system, cytokine signaling in immune system, virus infection, and signal transduction. Overall, there were multiple pathways, including immunology, viral infection, signal transduction, cancer, signaling by interleukins, TNF signaling pathway, advanced glycation end products (AGE)-receptor for advanced glycation end products (RAGE) signaling pathway in diabetic complications, IL-17 signaling pathway, Chagas disease (American trypanosomiasis), apoptosis, and hypoxia-inducible factor (HIF)-1 signaling pathway, found to be associated with the 434 curated gene sets of NRICM101–human genes–COVID-19. In alignment with the results of this study, a previous in silico study found that NRICM101 was related to pathways associated with cytokines and immune cells. Protective effects on TNF-a/IL-1b-induced cell injury were also demonstrated in the in vitro assay [12]. In addition, the dysregulation of the angiotensin (Ang) II-AT1 receptor (AT1R) pathway was found to be associated with cytokine release syndrome, indicating the potentiality of the cytokine signaling associated pathway as the therapeutic markers in COVID patients [17,18,19]. Thus, the pharmacogenomic analyses as well as the constructed dose–response profiles of IL-6 and TNF-α in this study could provide essential information for individual therapies or appropriate dose selections in more clinical treatments.

Notably, we also identified associations and interactions among different pathways based on the ontology enrichment clustering network. Pathways such as cytokine signaling in immune system, gastrin signaling pathway, lipid and atherosclerosis, apoptosis, pathways in cancer, AGE-RAGE signaling pathway in diabetic complications, and cellular response to nitrogen compound were more clustered. Other clusters, including pathways of cell activation, regulation of cell adhesion, regulation of defense response, and positive regulation of cell motility, were found to be linked with the cluster containing the cytokine signaling in immune system, which was in accordance with next-generation sequence analysis, showing that NRICM101 was associated with pathways of T-helper 1 (Th1)/T-helper 2 (Th2) immuno-regulation and cell movements of macrophages [12].

Complexities of infectious diseases are mainly ascribed to the intricate interactions among sets of genes and proteins involved in the process. Protein interactions could be visualized by networks created by complex interaction mappings, such as the protein–protein interaction (PPI) network and network pharmacology of herbs–chemicals–targets, which have gradually gained importance in addressing the complexities of COVID-19 disease onsets in the human body [20]. The generated PPI networks with the 30 most frequently curated genes/proteins of 434 gene sets were analyzed by the STRING database. There were 6 nodes with degree over 10 as hub genes, namely, RELA proto-oncogene, NF-κB subunit (RELA), signal transducer and activator of transcription 3 (STAT3), interleukin 6 (IL6), tumor protein P53 (TP53), mitogen-activated protein kinase 3 (MAPK3) and mitogen-activated protein kinase 1 (MAPK1). In alignment with the findings in this study, the aberrant STAT pathway was found to be central to COVID-19, and the acute lung injury also activated EGFR, leading to the phosphorylation of STAT3 [21].

Notably, the Huashi Baidu formula (HSBDF), which is another TCM found to be a clinically effective treatment for acute lung injury, has two herbs that are the same as those in NRICM101, which are Magnolia Bark and Baked Licorice Root [22]. In addition, 14 of the 30 most frequently curated genes of NRICM101 were found to be the same as the core target genes of HSBDF [23]. Additionally, similar to the results in this study, four identical hub genes, including IL6(12), TP53(12), MAPK3(11), and MAPK1(10), were also found to be core targets of TCM for the treatment of COVID-19 [24].

In this study, the disease analysis of CTD was classified by the Medical Subject Heading (MeSH) IDs, which are primary analytical elements extracted from the literature with calculated correlations [25]. The disease analyses were performed by using the gene set analyzer such that 30 of the most frequently gene sets were screened for investigations and applications of the STRING database in disease separations based on Disease Ontology (DOID) [26]. The major diseases based on the most curated NRICM101 chemicals and COVID-19 related genes were found to be vascular diseases (MESH: D014652, DOID: 178), gastrointestinal diseases (MESH: D005767, DOID: 77), cerebrovascular disorders (MESH: D002561, DOID: 6713), respiratory tract diseases (MESH: D012140, DOID: 11162 and DOID: 11394), metabolic diseases (MESH: D003920, DOID: 4194), hypertension (MESH: D006973, DOID: 10763), and immune system diseases (MESH: D007154, DOID: 2914).

Therefore, based on the results of disease analyses in this study, vascular diseases are the most correlated with the curated chemical of NRICM101 and COVID-19 associated genes. Therapeutic methods to address vascular system dysfunctions or related sequelae in COVID patients should be emphasized [27]. This study also found the correlation of acute cerebrovascular disease (CVD) with COVID-19. Since older patients with risk factors are more likely to develop CVD, this disease category could be used as an important negative prognostic factor to identify the optimal strategy in the prevention of the COVID-19 pandemics [28]. In addition, in accordance with the findings in disease analyses, although COVID-19 is primarily considered as a respiratory disease, accumulating evidence have shown its effects on the digestive system and the corresponding gastrointestinal (GI) symptoms [29]. Moreover, diabetes mellitus was one of the major disease candidates related with NRICM101-curated chemicals and COVID-19-associated genes. Supporting evidence showed that COVID-19 was capable of causing direct damages to the pancreas and worsening hyperglycemia, leading to inductions of diabetes onsets in non-diabetic patients [30].

Furthermore, it was reported that COVID-19 was associated with altered expressions of a few plasma microRNA (miRNAs), with miR-1275 and miR-31-5p being the most strongly down-regulated and up-regulated ones [31]. Interactions between miRNA and its target mRNA result in bound mRNA degradations, thereby repressing the replication. This mechanism is applicable in particular COVID-19 infections where viral genome is targeted and inhibited by the “matched” miRNA once it is released from the nucleus, leading to the suppression of multiplication and survival of the COVID-19 virus [32]. The mode of actions of some TCMs (e.g., honeysuckle) in their antiviral activities are also in accordance with the miRNA mechanism by inhibiting viral replications [33]. Moreover, TCM could offer unique advantages in preventing drug resistances caused by viral RNA sequence mutation, a common phenomenon often seen in some antiviral drugs. As a matter of fact, it is crucial to broadly understand the role of cellular miRNAs and miRNA-mediated gene silencing for COVID-19 diseases as a new option for therapeutic developments [34].

Taken together, based on the fact that NRICM101 acts as one of the clinically effective TCMs, being approved for emergent use in many countries, it is crucial to obtain the pharmacogenomic profile illustrating the interactions of its chemical contents with SARS-CoV-2 and human genes. The thorough network pharmacology and pathway/disease analyses based on the 434 gene sets from curated analyses of NRICM101 and COVID-19-related genes/proteins could also provide essential information for appropriate drug treatments with different gene profiles. More detailed mechanisms or modes of actions of NRICM101 components on gene or protein interactions, signaling pathways, and related disease symptoms should be deeply explored [16]. Not limited to clinical or bedside-to-bench study, this study could provide a well-constructed framework for investigations of pharmacogenomics and pharmacodynamics of TCMs. The pharmacogenomic information of NRICM101 provided in this study would also be essential when treating individuals with different gene profiles or be applicable in combination with other pharmaceutical administrations.

## 4. Materials and Methods

### 4.1. Curated Interaction Analysis

This study adopted the Comparative Toxicogenomics Database (CTD; Department of Bioinformatics, The Mount Desert Island Biological Laboratory, Salisbury Cove, ME, USA; http://ctd.mdibl.org (accessed on 12 June 2022)) to analyze curated interactions of NRICM101 and COVID-19. The CTD has powerful resources to explore interactions between chemical exposures and biological responses [35]. It is also capable of providing information including chemical–gene and/or chemical–protein interaction, chemical–disease and gene–disease relationships. The toxicogenomic data curated from the CTD include over 2,561,046 interactions among 14,311 chemicals and 53,648 genes from 622 species. There were also 41,383 gene/protein–disease direct relationships and 226,954 chemical–disease direct relationships found as of June 2022.

The chemical contents of the NRICM101 were analyzed based on the 2 TCM databases of BATMAN-TCM and TCM Database@Taiwan [36,37]. The BATMAN-TCM is an open database that can be accessed by inputting herbal contents of the interested TCM [35]. The TCM Database@Taiwan serves as the world’s largest TCM database for in silico drug screening and computer-aided drug design [37]. Information of COVID-19 related gene/protein was obtained from the CTD and Human Gene Database (GeneCards) [38] (https://www.genecards.org/ (accessed on 17 April 2022)). The GeneCards is an integrative database providing comprehensive information on human genes based on gene-centric data from ~150 web sources of genomic, transcriptomic, proteomic, genetic, clinical and functional information (https://www.genecards.org/ (accessed on 17 April 2022)).

There were 7522 chemical-related human genes analyzed from the BATMAN-TCM (blue color) and 7483 chemical-related human genes based on the TCM database@TW (red color) within the CTD. A total of 9884 co-targeted genes/proteins as targets of both NRICM101 and COVID-19 were found from the CTD (yellow color). Additionally, there were 1043 COVID-19 related genes/proteins based on the analyses of the Human Gene Database (GeneCards) (green color). Finally, 434 genes were selected from the interaction among the intersection of the four colored areas. Thus, the 434 COVID-19 related genes were selected based on the curated interaction analyses by using the CTD and GeneCards databases.

### 4.2. Gene Ontology, Pathway and Network Analyses

The interacting genes selected based on the curated interaction analysis of BATMAN-TCM and TCM Database@Taiwan databases, CTD, and GeneCards were comprehensively investigated with STRING to obtain pharmacogenomic analyses of GeneOntology (GO), pathways, networks, and related human diseases. Curated interactions of NRICM101 chemicals and interacting genes in human dataset were manually processed in the following steps: (1) curated interactions with chemicals of NRICM101 were all analyzed, (2) results of interactions with “No effect” were removed, and (3) networks of the protein–protein interaction (PPI) were established and visualized for intersected targets based on the STRING database [39]. In addition, the enrichment analyses, including gene ontology (GO), Kyoto Encyclopedia of Genes and Genomes (KEGG) and Reactome (REACT) pathways, were implemented. ShinyGO 0.76 [40], Metascape [41] and Cytoscape 3.9.1 [42] were used to implement and render the visualization, and pathways associated with COVID-19 pathogenesis were also manually integrated.

### 4.3. Disease Analysis

To establish disease relationships in the CTD, two kinds of resources were utilized, which were information of curation of chemical–disease and gene–disease relationships based on literature explorations and adoptions of gene–disease relationships from the database of the Online Mendelian Inheritance in Man (OMIM; McKusick-Nathans Institute of Genetic Medicine, Johns Hopkins University School of Medicine, MD, USA; http://www.ncbi.nlm.nih.gov/omim (accessed on 12 June 2022)). The OMIM is a freely available, comprehensive, and authoritative compendium of human genes and genetic phenotypes that is updated daily. Subsequently, potential human diseases were inferred from the NRICM101–gene–disease relationships based on the CTD and STRING databases [39].

### 4.4. Identifications of Target Genes

Since the role of miRNA in acting as a biomarker or therapeutic option for distinguishing COVID-19 infection from influenza infection has been found in several studies [31,34], there is also a viewpoint that the interplay of miRNA between host and SARS-CoV-2 could serve as the primary cause for SARS-CoV-2 accessing and attacking the host cells [43]. Thus, we are interested in finding potential targets of miRNA in patients treated with NRICM101 to provide a more detailed picture in the NRICM101–gene–disease relationship of pharmacogenomic analyses.

Predictions of targets of microRNA (miRNA) were performed by applying the miRabel database which aggregated all human results based on four important miRNA target prediction algorithms (miRanda, PITA, SVmicrO, and TargetScan) (LITIS lab, University of Rouen Normandy, France; http://bioinfo.univ-rouen.fr/mirabel/ (accessed on 12 June 2022)) [14]. The algorithms also showed additional characteristics for re-ranking miRNA targets in the platform.

### 4.5. Dose–Response Profile of NRICM101

Dose–response profiles of NRICM101 with different dilution folds and corresponding cytokine inhibitions (IL-6 or TNF-α expression) in cell cultures of murine alveolar macrophages were established by adapting the experimental data [10]. A three-parameter Hill model was applied to fit the experimental data describing relationship between dilution folds of NRICM101 and increments of IL-6 or TNF-α expression (%) [10]. Data values were presented as means of IL-6 or TNF-α expression in each dilution fold of NRICM101. The dose–response profiles characterizing the relationships between dilution (folds) and effects (*E*) in murine alveolar macrophages can be described as,

(1) $$E(D)=\frac{E_{\max}\cdot {\left(D_{}\right)}^{n}}{1+{\left(\frac{ED50}{D_{}}\right)}^{n}},$$

where *D* implies the dilution folds of NRICM101, *E*_max_ is the maximum responses of IL-6 or TNF-α expression (%), *ED*50 is the dilution fold corresponding to 50% *E*_max_, and *n* is the model-derived Hill coefficient, in which *n* = 1 means there is a Michaelis–Menten mode of linear response and *n* > 1 indicates that the biomarker is ultrasensitive to the effects of NRICM101 in the murine alveolar macrophages.

## 5. Conclusions

A systematic analysis was performed to explore the pharmacological mechanisms of NRICM101 by using bioinformatics and PD approaches. The computational approaches applied in this study could be applied to elucidate molecular mechanisms of chemicals in NRICM101 and related interacting genes/proteins with COVID-19. The 434 common human gene interactions were found based on overlapping analyses of NRICM101-related and COVID-19-associated genes. Disease analysis based on the NRICM101 chemical contents and interacting gene sets could also provide information of related mechanisms or modes of action for better treatment evaluations. Furthermore, the 30 most frequently curated human genes and 2 miRNAs can be used as molecular biomarkers to analyze the efficacy and mechanism of the NRICM101 in COVID-19 treatments. New markers for the early detection of viral infections, prognosis determination, and developments of TCM therapies based on detailed pharmacogenomic analyses are also revealed. This study thus provides the first thorough pharmacogenomic analysis of NRICM101 incorporated with the PD analyses such that the underlying drug–gene or drug–protein mechanisms and molecular pathways associated with disease onsets were explored. The information provided in this study could be further applied in the comparison of efficacies or safety among various TCMs and COVID-19 drugs.

## Figures and Tables

**Table 1:** The TCM formula, Taiwan Chingguan Yihau (NRICM101), including 10 herbs, has been used in Taiwan to treat COVID-19 patients since April 2020 [10].

Herbs	Binomial Name	Category	Weight (g)
Scutellaria Root	*Scutellaria baicalensis*	Heat-clearing and damp-drying medicine	18.75
Heartleaf Houttuynia	*Houttuynia cordata*	Heat-clearing and detoxifying drugs	18.75
Mongolian Snakegourd Fruit	*Trichosanthes kirilowii*	Heat-clearing phlegm medicine	18.75
Indigowoad Root	*Isatis indigotica*	Heat-clearing and detoxifying drugs	18.75
Magnolia Bark	*Magnolia officinalis*	Dampness medicine	11.25
Peppermint Herb	*Mentha haplocalyx*	Diffuse wind-heat medicine	11.25
Fineleaf Nepeta	*Nepeta tenuifolia*	Traditional Chinese Medicine	11.25
Mulberry Leaf	*Morus alba*	Traditional Chinese Medicine	11.25
Saposhnikovia Root	*Saposhnikovia divaricata*	Traditional Chinese Medicine	7.50
Baked Licorice Root	*Glycyrrhiza glabra*	Invigorating Qi Medicine	7.50

**Table 2:** The 30 most frequently curated NRICM101 chemicals and COVID-19 related genes.

Gene Symbols	Count@CTD	Gene Names	Location
TNF	298	tumor necrosis factor	6p21.33
CASP3	178	caspase 3	4q35.1
IL1B	157	interleukin 1 beta	2q14.1
INS	92	insulin	11p15.5
AKT1	89	AKT serine/threonine kinase 1	14q32.33
BAX	81	BCL2 associated X, apoptosis regulator	19q13.33
BCL2	79	BCL2 apoptosis regulator	18q21.33
NFE2L2	76	NFE2 like bZIP transcription factor 2	2q31.2
IL6	74	interleukin 6	7p15.3
CXCL8	71	C-X-C motif chemokine ligand 8	4q13.3
AHR	70	aryl hydrocarbon receptor	7p21.1
RELA	69	RELA proto-oncogene, NF-κB subunit	11q13.1
PTGS2	66	prostaglandin-endoperoxide synthase 2	1q31.1
MAPK3	61	mitogen-activated protein kinase 3	16p11.2
MAPK1	58	mitogen-activated protein kinase 1	22q11.22
TP53	58	tumor protein p53	17p13.1
PARP1	52	poly(ADP-ribose) polymerase 1	1q42.12
HMOX1	52	heme oxygenase 1	22q12.3
AR	47	androgen receptor	Xq12
HIF1A	45	hypoxia inducible factor 1 subunit alpha	14q23.2
STAT3	43	signal transducer and activator of transcription 3	17q21.2
VEGFA	37	vascular endothelial growth factor A	6p21.1
TGFB1	36	transforming growth factor beta 1	19q13.2
CCL2	36	C-C motif chemokine ligand 2	17q12
CCND1	35	cyclin D1	11q13.3
CASP8	35	caspase 8	2q33.1
ALB	32	albumin	4q13.3
TLR4	31	toll like receptor 4	9q33.1
CAT	29	catalase	11p13
ABCC1	29	ATP binding cassette subfamily C member 1	16p13.11

**Table 3:** Gene Set Analyzer: Top 30 enriched diseases from the 30 most frequently curated NRICM101 chemicals and COVID-19 related genes. (Threshold: corrected *p* value < 0.01, with all disease category).

Disease Name	Disease ID	Disease Categories	*p*-Value	Corrected *p*-Value	Annotated Genes Quantity	Genome Frequency
Vascular Diseases	MESH:D014652	Cardiovascular disease	9.56 × 10^−48^	1.05 × 10^−44^	29	966/44,746 genes: 2.16%
Cardiovascular Diseases	MESH:D002318	Cardiovascular disease	6.11 × 10^−45^	6.71 × 10^−42^	30	1517/44,746 genes: 3.39%
Neoplastic Processes	MESH:D009385	Cancer|Pathology (process)	2.66 × 10^−44^	2.92 × 10^−41^	25	516/44,746 genes: 1.15%
Gastrointestinal Diseases	MESH:D005767	Digestive system disease	1.24 × 10^−43^	1.36 × 10^−40^	28	1072/44,746 genes: 2.40%
Heart Diseases	MESH:D006331	Cardiovascular disease	3.07 × 10^−43^	3.37 × 10^−40^	28	1107/44,746 genes: 2.47%
Endocrine System Diseases	MESH:D004700	Endocrine system disease	1.70 × 10^−42^	1.86 × 10^−39^	28	1176/44,746 genes: 2.63%
Cerebrovascular Disorders	MESH:D002561	Cardiovascular disease|Nervous system disease	2.57 × 10^−42^	2.82 × 10^−39^	21	224/44,746 genes: 0.50%
Male Urogenital Diseases	MESH:D052801	Urogenital disease (male)	6.58 × 10^−42^	7.23 × 10^−39^	29	1528/44,746 genes: 3.41%
Diabetes Mellitus	MESH:D003920	Endocrine system disease|Metabolic disease	1.38 × 10^−41^	1.51 × 10^−38^	23	410/44,746 genes: 0.92%
Liver Diseases	MESH:D008107	Digestive system disease	2.08 × 10^−41^	2.29 × 10^−38^	30	1985/44,746 genes: 4.44%
Central Nervous System Diseases	MESH:D002493	Nervous system disease	5.49 × 10^−41^	6.03 × 10^−38^	28	1330/44,746 genes: 2.97%
Respiratory Tract Diseases	MESH:D012140	Respiratory tract disease	9.99 × 10^−41^	1.10 × 10^−37^	27	1101/44,746 genes: 2.46%
Lung Diseases	MESH:D008171	Respiratory tract disease	1.96 × 10^−40^	2.15 × 10^−37^	26	912/44,746 genes: 2.04%
Brain Diseases	MESH:D001927	Nervous system disease	7.75 × 10^−40^	8.51 × 10^−37^	27	1187/44,746 genes: 2.65%
Reperfusion Injury	MESH:D015427	Cardiovascular disease|Pathology (process)	1.71 × 10^−39^	1.88 × 10^−36^	19	169/44,746 genes: 0.38%
Gastrointestinal Neoplasms	MESH:D005770	Cancer|Digestive system disease	4.00 × 10^−39^	4.39 × 10^−36^	25	825/44,746 genes: 1.84%
Hypertension	MESH:D006973	Cardiovascular disease	7.60 × 10^−39^	8.35 × 10^−36^	20	245/44,746 genes: 0.55%
Postoperative Complications	MESH:D011183	Pathology (process)	8.40 × 10^−39^	9.23 × 10^−36^	19	183/44,746 genes: 0.41%
Pathologic Processes	MESH:D010335	Pathology (process)	2.37 × 10^−38^	2.61 × 10^−35^	30	2506/44,746 genes: 5.60%
Genital Diseases	MESH:D000091662		3.40 × 10^−38^	3.73 × 10^−35^	27	1364/44,746 genes: 3.05%
Female Urogenital Diseases	MESH:D052776	Urogenital disease (female)	6.01 × 10^−38^	6.60 × 10^−35^	27	1393/44,746 genes: 3.11%
Urogenital Diseases	MESH:D000091642		1.16 × 10^−37^	1.27 × 10^−34^	29	2136/44,746 genes: 4.77%
Nervous System Diseases	MESH:D009422	Nervous system disease	2.15 × 10^−37^	2.36 × 10^−34^	30	2696/44,746 genes: 6.03%
Digestive System Neoplasms	MESH:D004067	Cancer|Digestive system disease	2.28 × 10^−37^	2.50 × 10^−34^	27	1463/44,746 genes: 3.27%
Wounds and Injuries	MESH:D014947	Wounds and injuries	2.48 × 10^−37^	2.73 × 10^−34^	19	217/44,746 genes: 0.48%
Cardiomyopathies	MESH:D009202	Cardiovascular disease	2.87 × 10^−37^	3.15 × 10^−34^	20	292/44,746 genes: 0.65%
Lung Neoplasms	MESH:D008175	Cancer|Respiratory tract disease	5.63 × 10^−37^	6.18 × 10^−34^	23	645/44,746 genes: 1.44%
Respiratory Tract Neoplasms	MESH:D012142	Cancer|Respiratory tract disease	6.74 × 10^−37^	7.40 × 10^−34^	23	650/44,746 genes: 1.45%
Thoracic Neoplasms	MESH:D013899	Cancer	6.99 × 10^−37^	7.67 × 10^−34^	23	651/44,746 genes: 1.45%
Digestive System Diseases	MESH:D004066	Digestive system disease	8.26 × 10^−37^	9.07 × 10^−34^	30	2819/44,746 genes: 6.30%

**Table 4:** The potential target miRNAs of the 30 most frequently curated genes were predicted using the miRabel database.

hsa-miR-31-5p								
Gene	miRabel Score	PITA	miRanda	SVMicrO	TargetScan	ExpVal	5′UTR	CDS
CXCL8	0.992007	3689	-	-	-	NO	NO	YES
TGFB1	0.973495	4950	5760	-	-	NO	NO	NO
STAT3	0.967355	5261	5014	-	-	NO	NO	YES
VEGFA	0.853542	3537	1675	-	-	NO	NO	NO
ALB	0.817103	1213	2922	-	-	NO	NO	YES
AHR	0.699523	6587	3055	-	2730	NO	NO	NO
MAPK1	0.37997	3026	2550	-	2199	NO	NO	NO
PARP1	0.211814	3328	424	-	1367	YES	NO	YES
TLR4	0.117578	151	727	-	2149	NO	NO	YES
AR	0.0587212	431	348	-	162	NO	NO	YES
**hsa-miR-1275**								
PARP1	0.996829	6499	-	10,247	-	NO	NO	YES
HMOX1	0.995508	5737	-	9682	-	NO	NO	YES
CASP8	0.99529	-	6015	7537	-	NO	NO	YES
AKT1	0.980752	7480	6376	5807	-	NO	NO	NO
CCL2	0.965806	588	-	7875	-	NO	NO	YES
MAPK3	0.948772	7059	3780	5233	-	NO	NO	YES
AHR	0.934906	6196	3884	6468	-	NO	NO	YES
MAPK1	0.916016	4397	-	6283	4585	NO	YES	NO
ABCC1	0.909767	2023	6924	5072	-	NO	NO	NO
RELA	0.788465	-	3043	14,573	2276	NO	NO	YES
VEGFA	0.712891	6277	6815	2039	-	NO	NO	NO
TP53	0.108736	13	305	2937	-	NO	NO	YES
STAT3	0.0711241	6311	1637	2321	2840	NO	NO	YES
TGFB1	0.0589265	4304	2364	1363	4383	NO	YES	NO

## Data Availability

Not applicable.

## Supplementary Materials

The following are available online at https://www.mdpi.com/article/10.3390/ijms232315385/s1.

## Abbreviations

TCM, traditional Chinese medicine; COVID-19, coronavirus disease; CTD, Comparative Toxicogenomics Database; PPI, protein–protein interaction.
