# Supplementary material for: Pharmacological Mechanism of NRICM101 for COVID-19 Treatments by Combined Network Pharmacology and Pharmacodynamics
Source: Int J Mol Sci. 2022 Dec 6;23(23):15385. doi: 10.3390/ijms232315385 (PMC9740625; doi:10.3390/ijms232315385)
Supplement: Supplementary file 1 [file ijms-23-15385-s001.zip › TableS11.pdf]

Table S11. Fitted coefficients (mean ± S.E.) of the three-parameter Hill model describing IL-6 or TNF-α expressions in murine alveolar macrophages treated with different dilution folds of the NRICM101 based on the experimental data adapted from [10].

	**IL-6**	**TNF-α**
E_{max} (%)	91.61 ± 4.31	93.58 ± 2.69
*EC*50 (folds)	93.31 ± 17.91	39.71 ± 4.13
n	1.00 ± 0.16	1.83 ± 0.30
r^2	0.98	0.98
p-value	***	***

*** $p < 0.001$.

Reference

10. Tsai, K.-C.; Huang, Y.-C.; Liaw, C.-C.; Tsai, C.-I.; Chiou, C.-T.; Lin, C.-J.; Wei, W.-C.; Lin, S. J.-S.; Tseng, Y.-H.; Yeh, K.-M.; Lin, Y.-L.; Jan, J.-T.; Liang, J.-J.; Liao, C.-C.; Chiou, W.-F.; Kuo, Y.-H.; Lee, S.-M.; Lee, M.-Y.; Su, Y.-C., A traditional Chinese medicine formula NRICM101 to target COVID-19 through multiple pathways: A bedside-to-bench study. *Biomedicine & Pharmacotherapy* **2021,** 133, 111037.
